# A Linear Approach to Optimize an EMG-Driven Neuromusculoskeletal Model for Movement Intention Detection in Myo-Control: A Case Study on Shoulder and Elbow Joints

**DOI:** 10.3389/fnbot.2018.00074

**Published:** 2018-11-13

**Authors:** Domenico Buongiorno, Michele Barsotti, Francesco Barone, Vitoantonio Bevilacqua, Antonio Frisoli

**Affiliations:** ^1^Department of Electrical and Information Engineering, Polytechnic University of Bari, Bari, Italy; ^2^Percro Laboratory, Tecip Institute, Scuola Superiore Sant'Anna, Pisa, Italy

**Keywords:** neuromusculoskeletal model, EMG, upper limb, optimization, myo-control, genetic algorithm, torque prediction, exoskeleton

## Abstract

The growing interest of the industry production in wearable robots for assistance and rehabilitation purposes opens the challenge for developing intuitive and natural control strategies. Myoelectric control, or myo-control, which consists in decoding the human motor intent from muscular activity and its mapping into control outputs, represents a natural way to establish an intimate human-machine connection. In this field, model based myo-control schemes (e.g., EMG-driven neuromusculoskeletal models, NMS) represent a valid solution for estimating the moments of the human joints. However, a model optimization is needed to adjust the model's parameters to a specific subject and most of the optimization approaches presented in literature consider complex NMS models that are unsuitable for being used in a control paradigm since they suffer from long-lasting setup and optimization phases. In this work we present a minimal NMS model for predicting the elbow and shoulder torques and we compare two optimization approaches: a linear optimization method (LO) and a non-linear method based on a genetic algorithm (GA). The LO optimizes only one parameter per muscle, whereas the GA-based approach performs a deep customization of the muscle model, adjusting 12 parameters per muscle. EMG and force data have been collected from 7 healthy subjects performing a set of exercises with an arm exoskeleton. Although both optimization methods substantially improved the performance of the raw model, the findings of the study suggest that the LO might be beneficial with respect to GA as the latter is much more computationally heavy and leads to minimal improvements with respect to the former. From the comparison between the two considered joints, it emerged also that the more accurate the NMS model is, the more effective a complex optimization procedure could be. Overall, the two optimized NMS models were able to predict the shoulder and elbow moments with a low error, thus demonstrating the potentiality for being used in an admittance-based myo-control scheme. Thanks to the low computational cost and to the short setup phase required for wearing and calibrating the system, obtained results are promising for being introduced in industrial or rehabilitation real time scenarios.

## 1. Introduction

In the last decades the interest in powered exoskeleton suits has known a substantial growth (Hill et al., 2017). Exoskeletons are “wearable robots” conceived for being worn by the users in order to assist or increase their physical performance (Wolff et al., 2014; Buongiorno et al., 2018). This kind of devices are mostly exploited in industrial applications, with the aim of decreasing workers' pain and preventing musculoskeletal disorders (Bostelman et al., 2017; de Looze et al., 2017). They are also used *in (neuro)*rehabilitation applications with the aim of enhancing the recovery process and minimizing functional disability, with consequent earlier reintegration in activities of daily living (Frisoli et al., 2016; Veerbeek et al., 2017).

Theoretically, *myoelectric control* (or *myo-control*) represents a straightforward way to decode the human motor intent from electromyographic signals (EMG) and encode it into high-level input signals for controlling exoskeletons or prosthesis (Leonardis et al., 2015; Farina et al., 2017; Vujaklija et al., 2017). Despite the technology behind the development of exoskeletons is rapidly growing, the myo-control schemes have yet to be validated as viable control approach for commercial applications in robotic exoskeletons control (Ison and Artemiadis, 2014). Indeed, the myo-control schemes based on pattern recognition are not suitable for being used in rehabilitation or industry applications due to both their inability to simultaneously activate several degrees of freedom (DoFs) and the low classification accuracy (Hahne et al., 2017). In addition, pattern recognition approaches do not allow to modulate the level of robotic assistance resulting in a non-natural user-exoskeleton interaction. As example, a trigger-based control scheme allows the user to start a predefined robot movement without being able of controlling the speed or the direction of the movement once it is started (Barsotti et al., 2015). It follows that simultaneous and proportional control paradigms have been gaining attention over the last years (Jiang et al., 2012; Buongiorno et al., 2015, 2016, 2017; Lobo-Prat et al., 2017), establishing themselves as promising tools to reduce the gap between research and commercial applications (Roche et al., 2014).

Among the myo-control schemes, the ones relying on the biological aspects of the human control have revealed to be the most suitable for real scenario applications (Aoi and Funato, 2016; Crouch and Huang, 2017). The two main “*biologically inspired*” schemes for myo-control are: the synergistic approach and the model-based approach. Although the debate about whether the human motor system composes complex movements through flexible combinations of elementary bricks (also called *muscle synergies*) or not (Kutch and Valero-Cuevas, 2012; Berger et al., 2013), several studies investigated the synergistic approach for myoelectric control focusing on the estimation of the force applied by the hand (Berger and d'Avella, 2014; Buongiorno et al., 2017). The *model-based* paradigms, that uses EMG-driven neuromusculoskeletal (NMS) models, are particularly suited for the estimation of the human's articulation moments (Lloyd and Besier, 2003; Buchanan et al., 2004; Sartori et al., 2012, 2015), thus theoretically representing a valid solution for motor intention detection in myo-control. On the other hand, such detailed EMG-driven bio-mechanical models require long lasting setup and calibration procedures since they consider (1) the muscle activity of a large number of muscles and (2) a big set of model's parameters that has to be optimized (Durandau et al., 2018). These aspects lead to a low usability of the model-based myo-control scheme in a real application scenario, of course.

Several studies in literature demonstrated the ability of accurately estimating the joints torques of high detailed bio-mechanical models which require long lasting setup and calibration procedures (Durandau et al., 2018). Lloyd et al. proposed a detailed model of the knee composed by 15 muscles and adjusted 4 parameters for each muscle without considering the optimization of the NMS model geometry (Lloyd and Besier, 2003). Sartori et al. built a sophisticated knee joint model with 13 muscles, and they found that on the basis of the model complexity the optimization procedure could last from few minutes to 5 h (Sartori et al., 2012). Buchanan et al. modeled and tested a human elbow with seven primary muscles during isometric contractions (Buchanan et al., 2004). Although Buchanan et al. considered many muscles, they optimized only four EMG-dependent parameters without considering the NMS geometry optimization. In Pau et al. (2012) the authors used a two-muscles elbow's model (24 parameters in total) to estimate the elbow's movements. The optimization procedure, based on a genetic algorithm, featured of an execution time of about an hour. Cavallaro et al. (2006) presented an EMG-driven NMS model with a large set of optimized parameters (11 per muscle) to estimate the elbow and the wrist moments from the activity of 12 muscles. The optimization was based on a genetic algorithm - optimization time is not reported. Fleischer et al. proposed a simplified model of the knee composed by six muscles (Fleischer and Hommel, 2008) and optimized 4 parameters per muscle. The model presented by Fleischer et al. has then been used to control a lower limb exoskeleton.

From the analysis of the literature, it then emerged that really few research groups (Fleischer and Hommel, 2008; Pau et al., 2012) have so far investigated the performance of simplified versions of a EMG-driven NMS model to reduce both the setup and calibration phases. It might be thought that the lack of research works investigating the use of EMG-driven models for the online control of upper limb devices is explained by: (1) the shortage of simple optimization approaches, and (2) the absence of study that validate and discuss the performance of model composed by a reduced number of muscles.

Given the demonstrated ability of NMS model in predicting joints torques, in this work we propose a minimal EMG-driven NMS model of the elbow and shoulder joints for moment prediction, and we present a comparison between a simple linear optimization procedure (LO) vs. a more complex one based on a genetic algorithm (GA-based). In addition to the algorithm complexity, the two procedures differ also in the number of optimized model's parameters: the GA-based method optimizes 12 parameters per muscle, whereas the linear method adjusts only 1 parameter per muscle. In this way it is possible to compare a really fast approach (LO) which optimizes only the basic model parameters (i.e., body mass and maximum muscle's force) against one which takes into account a bigger set of the muscle variables and non-linear relationships (GA-based). This manuscript extends our previous works (Buongiorno et al., 2015, 2016) under three main aspects: increasing the number of tested subjects for both methods, extending the investigation of the LO method from the elbow joint only to both the elbow and shoulder joints and, reporting a structured comparison between the two methods. It is worth mentioning that both methods have been applied on a simplified upper limb model featuring only four muscles for the shoulder and two muscles for the elbow. Hence, the proposed model has the main advantage of requiring a short time for both the setup and the calibration phases making such approach suitable for being introduced real applications.

In the sections that follow, we present the experimental setup, the adopted EMG-driven NMS model, the description and the comparison of the two proposed optimization methods. We present the data acquisition protocol and the experimental setup, the adopted EMG-driven NMS model, and the description of the two optimization approaches in the second section. In the third section, we report the results of the comparison study including the statistical analysis. Finally, we discuss the main findings of the study correlating them to the industrial and medical applications.

## 2. Materials and methods

We asked participants to wear an upper limb exoskeleton and reach force-targets in a virtual environment provided by means of a head-mounted display (HMD). The human hand was rendered as a virtual spherical object moving accordingly to the *isometric* force applied to the exoskeleton's handle provided with a 3-axis force transducer.

### 2.1. Participants

Seven healthy, right-handed male subjects (mean age 26.7 years, SD 2.6, age range 23–30) participated in the experiments after giving written informed consent. The experimental procedures were conducted in accordance with the Declaration of Helsinki and approved by the Ethical Review Board of Scuola Superiore Sant'Anna. All except one subject had no previous experience with upper-limb exoskeleton interaction.

### 2.2. Experimental setup

**Figure 1:**
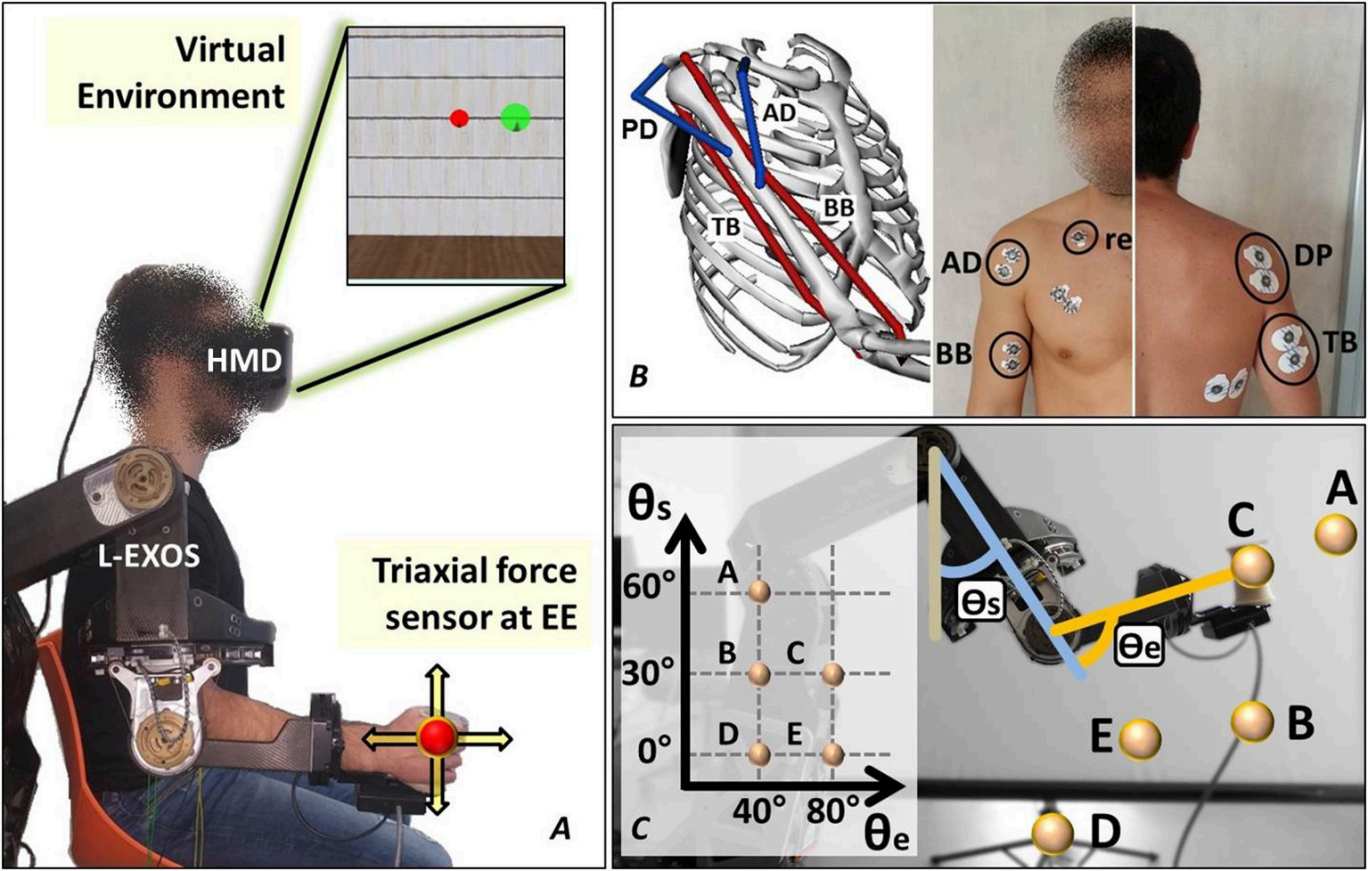
The experimental setup. **(A)** The subject wears the upper limb exoskeleton controlled in position and the HMD displaying the cursor and the target spheres in a 3D virtual environment. **(B)** The neuromusculoskeletal model adopted in the study and the electrodes placement. **(C)** The five end-effector positions lying on the sagittal plane explored in the experiment.

Subjects were asked to wear the upper limb exoskeleton Light-Exoskeleton (L-Exos, Frisoli et al., 2005) on the right arm (Figure 1A) and grab the sensorized handle (or end-effector, EE). Subjects' arm was then fixed to the exoskeleton's structure by means of an appositely conceived belt. The L-Exos has four actuated DoFs conceived for supporting elbow and shoulder movements (shoulder adduction/abduction, shoulder flexion/extension, shoulder internal/external rotation and elbow flexion/extension) and one passive DoF used for measuring the wrist prono-supination angle. Subjects were immersed in a virtual room characterized by only textured floor and walls by wearing a HDM (Oculus Rift, Oculus VR). The virtual engine displayed a spherical red cursor matching the position of the L-Exos's handle, thus providing the user with a visual perception of his real hand position (Figure 1A). Surface electromyographic (EMG) activity was recorded from the following four muscles acting on the shoulder and elbow: biceps brachii long head (BB), triceps brachii long head (TB), anterior deltoid (AD), and posterior deltoid (PD) (Figure 1B). These four muscles have been selected since BB and TB features the higher moment generation capacity among all the muscle crossing the elbow joint (Murray et al., 2000), whereas AD and PD are the most activated muscles during shoulder flexion and extension (Kronberg et al., 1990). EMG activity was recorded with passive Ag/AgCl foam pre-gelled electrodes with a diameter of 24 mm placed in bipolar configuration (inter-electrode distance set to 20 mm). All electrodes were connected to the g.USBamp amplifier, (http://www.gtec.at/) and digitally converted (1,200 Hz sample frequency, 12 bit resolution). The amplifier provided a high SNR built-in digital filter (5 –500 Hz) and the notch filter was set at 50 Hz. Ground and reference electrodes were positioned on the right clavicle. All the measurements were conducted following SENIAM recommendations (Hermens et al., 1999). The L-Exos's pose (joint angles) and the force applied to the EE were also recorded with a sample frequency of 1,200 Hz. The virtual scene was designed using the XVR software (VRMedia) and rendered by a PC workstation with a refresh rate of 60 Hz. The position of the virtual cursor was computed in real time using a simple spring model that elaborates the 20 Hz low-pass filtered force data.

### 2.3. Data acquisition protocol

The acquisition session started with the recording of the EMG data corresponding to the maximum voluntary contraction (MVC). The subject is seated in front of a desktop with the his right elbow flexed at 90 deg and fixed to the desktop itself, and is asked to apply in sequence the maximum force against the desktop along four directions (upward, downward, frontward and rearward respect to the desktop) and maintain the maximum contraction for 10 s. The subject, after 10 min break, was invited to wear both the exoskeleton and the HMD and grasp the exoskeleton's handle while supporting his arm. Once the subject is immersed in the virtual environment, he was asked to perform isometric reaching tasks following the instructions shown in the virtual scenario. In particular, the tasks consisted in moving a virtual cursor (a red semi-transparent sphere) from the rest position (i.e., zero force) to the target position (a green semi-transparent sphere) by applying isometric force at the EE (see Figure 1A). During the acquisition protocol, the subject has to reach 8 randomly assigned targets (2 targets × 4 directions) in each of five different positions of his right hand. The five predefined hand's positions lie on a plane that is parallel to the sagittal plane and crosses the shoulder articulation (see Figure 1C-the authors will refers to such plane as “sagittal plane”). The distance between the target and the rest cursor position was set such that the module of the force needed to reach the target was equal to 15 N. In order to provide a more natural interaction, the cursor position was not restricted in the sagittal plane. A trial consisted in the reaching of a single target. At the beginning of each trial, the subject is requested to keep the red cursor still for 2 s. Next, a green target sphere appears and subject had to reach the target and keep the cursor into the target for 0.2 s. The admitted force error is set to 2 N (proportional to the difference between the radius of the target sphere and the radius of the cursor sphere). Once the task was successfully completed the force target disappeared and the subject was asked to return in the rest position for ending the trial.

The adopted strategy to drive the robotic exoskeleton at the five experimental poses is based on the bounded jerk on-line trajectory planning proposed in Frisoli et al. (2013). A proportional-derivative joint position control with a feed-forward gravity compensation was used to both control the robot pose during the movements among the five poses and to guarantee the isometric condition during each trial. The control torque vector of the L-Exos **τ_*J*_** (4 × 1) is defined at joint level by the following equation:

(1) $$\begin{array}{l} \tau_{J}=K_{P}(\theta^{*}-\theta )+K_{D}({\dot{\theta }}^{*}-\dot{\theta })+\tau_{G}(\theta ) \end{array}$$

where θ^*^ and θ are the vectors (4 × 1) of the desired and current joint angles, respectively; $\dot{\theta^{*}}$ and $\dot{\theta }$ are the vectors (4 × 1) of the desired and current estimated joint speeds, respectively; ***K_P_*** and ***K_D_*** the diagonal positive-definite proportional and derivative gain matrices (4 × 4), respectively. To compensate the mass of each moving links of the robotic exoskeleton, the feedforward compensation term, **τ_*G*_**(θ), based on the kinetostatic model of the robot, was provided.

### 2.4. The adopted EMG-driven hill-based NMS model

In order to shorten the time needed for the system calibration and setup, thus making the approach suitable for the potential clinical and industrial applications, we adopted a simplified version of the EMG-driven NMS model reported in Buchanan et al. (2004) and Lloyd and Besier (2003) (see Supplementary Material for a brief description of the model). In particular, we have introduced the following simplifications:

- the muscle activation dynamic was not considered since it is strongly dependent on both electrode placement and skin condition (Lloyd and Besier, 2003), in fact such a large variability (that has also been reported in Sartori et al., 2012) prevents the choice of a default (or not optimized) value for the recursive filter's parameters. This simplification was already introduced in Cavallaro et al. (2006).
- in order to minimize the level of detail of the model, the optimal muscle fiber length, *l*_*o*_ was considered independent from the muscle activation level. Lloyd et al. have demonstrated that such simplification on the knee joint leads just to a slight decrement of the moment estimation performance (from *R*^2^ = 0.91 to *R*^2^ = 0.85) (Lloyd and Besier, 2003).
- we considered a stiff-tendon model; this assumption is supported by Sartori et al. (2009, 2012), which determined that a stiff-tendon model do not compromise the model prediction ability. In particular in the work of Sartori et al. (2012) a stiff-tendon model introduced an error that, on average, was <10% of the range of variation observed in the reference values respect with an elastic-tendon model. However, the moments predicted with the stiff-tendon model were better correlated to the experimental moments.

Considering that the NMS model has been trained in isometric conditions (*v*(*t*) ≈ 0, ∀*t* that implies *f*_*v*_(*v*) ≈ 1 - see Supplementary Material for a detailed definition of *f*_*v*_(*v*)), it results that the adopted EMG-driven model could be described by the following equations:

(2) $$\begin{array}{l} \tau^{p}=\overset{N}{\underset{i=1}{\sum }}\tau_{i}=\overset{N}{\underset{i=1}{\sum }}F_{i}^{mt}r_{i}\left(\theta \right) \end{array}$$

(3) $$\begin{array}{l} F^{mt}=F^{t}=F^{m}\cos\phi =F_{O}^{m}\left[f_{l}\left(\tilde{l}\right)\cdot a+f_{P}\left(\tilde{l}\right)\right]\cos\phi \end{array}$$

(4) $$\begin{array}{l} \tilde{l}=\frac{l}{l_{o}},\;a\left(t\right)=\frac{e^{Au\left(t\right)}-1}{e^{A}-1},\;\phi =arcsin\frac{l_{o}\sin\phi_{o}}{l} \end{array}$$

(5) $$\begin{array}{l} u\left(t\right)=e\left(t-d\right) \end{array}$$

where τ^*p*^ is the predicted articulation moment, *N* is the number of muscles considered in modeled articulation, τ_*i*_ is the moment contribution of the i-th muscle-tendon unit, *r*(θ) is the moment arm that depends on the articulation angles, *F*^*mt*^ is the force generate by the muscle-tendon unit, *F*^*t*^ is the tendon force, *F*^*m*^ is the force generated by the muscle fibers; $F_{O}^{m}$ is the maximum isometric muscle fiber force; $f_{l}\left(\tilde{l}\right)$ is the normalized fiber length-force relationship; $f_{p}\left(\tilde{l}\right)$ is the normalized fiber length- passive force relationship; *a* is the muscle activation level; *l* and *v* are the fiber length and fiber contraction velocity, respectively; $\tilde{l}$ is the normalized fiber length; *l*_*o*_ is the optimal fiber length; ϕ_*o*_ is the pennation angle at *l*_*o*_. The parameter *A* identifies the non-linearity shape factor (*A* can range in [−3, 0] Lloyd and Besier, 2003)and *d* is the electro-mechanical delay (range from 10 to 150 ms Corcos et al., 1992). *e*(*t*) represents the pre-processed EMG signal obtained with the three consecutive following steps:

1. high-pass filtered (20 Hz second-order Butterworth);
2. rectified and normalized over the MVC;
3. low-pass filtered (5 Hz second-order Butterworth).

### 2.5. Model optimization procedures: genetic algorithm and linear approach

It is well-known that an optimization procedure is required for adapting the model to the specific subject, and thus improving the prediction of the joint moments. In this work the following two optimization procedures are proposed and compared:

1. a genetic algorithm based optimization procedure (GA-based);
2. a linear optimization procedure (LO).

Although both the GA-based and the LO approaches optimize the same EMG-driven NMS model (see section 2.4), the two methods are substantially different and consider the optimization of distinct sets of model's parameters (see Table 1). Since the arm weight affects the tonic component of the EMG signals, both the optimization procedures considered the gravitational model of the arm. As it will be deeply discussed below, both methods require also the knowledge of a detailed muscluloskeletal model that specifies the numerical value of all used parameters and the trends of all relationships. Table 1 reports the default (normative or not optimized) value/trend of the model parameters/curves which have been taken from different sources:

- the literature (e.g., muscle fiber length, optimal muscle fiber length, etc);
- measures of subject's body (e.g., bones' length and body mass);
- from experimental trials (e.g., the electromechanical delay).

**Table 1:** List of the adopted EMG-driven NMS model parameters.

**Parameter**	**Description**	**Default value**	**GA**	**LO**
**Muscle/Articulation Variables**				
(1) *x*	electromechanical delay	80 ms (experimentally set)	–	–
(2) *A*	non-linearity factor	−0.2 Sartori et al. (2012)	✓	–
(3) *l*_*o*_	optimal fibers length	from Holzbaur et al. (2005)	✓	–
(4) ϕ_*o*_	pennation angle at *l*_*o*_	from Holzbaur et al. (2005)	–	–
(5) $F_{O}^{m}$	maximum isometric force	from Holzbaur et al. (2005)	✓	✓
**Muscle/Articulation Relationships**				
(6) *l*^*m*^(θ)	fibers-length/articulation-angle	from Holzbaur et al. (2005)	✓	–
(7) $f_{A}\left(\tilde{l}\right)$	normalized active-force/fiber-length	from Holzbaur et al. (2005)	✓	–
(8) $f_{P}\left(\tilde{l}\right)$	normalized passive-force/fiber-length	from Holzbaur et al. (2005)	–	–
(9) *r*(θ)	moment arm/articulation-angle	from Holzbaur et al. (2005)	✓	–
**Arm Gravity Model Parameters**				
(10) *M*_*a*_	arm mass	percentage of body mass	✓	✓
(11) *M*_*fh*_	forearm/hand mass	percentage of body mass	✓	✓
(12) *L*_1_	arm length	measured	–	–
(13) *l*_1_	position of the arm's center of mass	half of the arm length	–	–
(14) *l*_2_	position of the forearm's center of mass	half of the forearm-hand length	–	–

*For each of the optimization procedure (GA-genetic algorithm and LO-linear optimization), the checkmark and the dash indicate that the parameter/curve has been optimized or not, respectively. The parameter values have been set as the default values in case the specific parameter was not considered in the optimization. The default values have been also used to define ranging intervals used by the genetic algorithm reported in Table 2*.

All the parameters/curves obtained from the literature have been extracted by the Upper-Limb MusculoSkeletal Model developed by Holzbaur et al. (2005) using the simulation software OpenSim (Delp et al., 2007).

#### 2.5.1. The arm gravity model

Here, we briefly report the expressions of the gravity moments at the shoulder and elbow articulations, $\tau_{S}^{g}$ and $\tau_{E}^{g}$, respectively. The two-dimensional gravity model is described by the following equations that are functions of the shoulder's elevation angle θ_1_ and elbow's flexion angle θ_2_:

(6) $$\begin{array}{l} \tau_{el}^{g}=M_{fh}\;\cdot \;l_{2}\;\cdot \;g\;\cdot \;\sin(\theta_{1}+\theta_{2})\\ \tau_{sh}^{g}=M_{fh}\;\cdot \;L_{1}\;\cdot \;g\;\cdot \;\sin(\theta_{1})+M_{a}\;\cdot \;l_{1}\;\cdot \;g\;\cdot \;\sin(\theta_{1})+\tau_{el}^{g} \end{array}$$

where *g* is the gravitational acceleration, the arm mass is indicated with *M*_*a*_, the forearm/hand mass with *M*_*fh*_, the length of the arm is *L*_1_ while *l*_1_ is the distance between the center of mass of the arm and the rotational joint axes of the shoulder and *l*_2_ is the distance between the center of mass of the forearm/hand system and the rotational joint axes of the elbow. The arm length (*L*_1_) was measured from the acromiale to radiale, whereas the forearm/hand system length is measured from the bony tip of the elbow to the tip of the middle finger. The distances *l*_1_ and *l*_2_ were set equal to the half of the arm length and to the half of the forearm plus hand length, respectively.

#### 2.5.2. Ga-based optimization procedure

A GA-based optimization procedure allows to overcome the difficulty of a non-linearity optimization problem (Menolascina et al., 2009). In order to reduce the computational complexity of the GA, we discarded the following four parameters from the optimization procedure:

1. the value of the *electromechanical delay*, that has been set to 80 ms to maintain the temporal synchronization between muscle activation signals and moment signals (as done in Lloyd and Besier, 2003). This value has not been optimized since it is affected by a minimal range variation (Cavanagh and Komi, 1979).
2. the *pennation angle* at the optimal fiber length has not been optimized as done in most of the previous studies (Lloyd and Besier, 2003; Buchanan et al., 2004; Sartori et al., 2012, 2015)
3. the *normalized passive-force/fiber-length* relationship. Since the exercise was designed in such a way that the subjects' muscles never reached elongated configurations, we considered negligible the force contribution of the muscle tissue elasticity.
4. the length of the considered body's links that have been measured.

The muscle/articulation relationships *l*^*m*^(θ) and *r*(θ), were fitted using a 3^*rd*^ order polynomial function. It results that four coefficients had to be optimized for each relationship ($a_{i}^{l}$ and $a_{i}^{r}$ indicate the i-th coefficient of *l*^*m*^(θ) and *r*(θ), respectively). The active force/fibers-length $f_{A}\left(\tilde{l}\right)$ has been fitted with a Gaussian function with unitary mean and the standard deviation σ_*A*_ as the optimized parameter. The fitting procedures were conducted using the least squares approach, obtaining an averaged adjusted *R*^2^ coefficient of 0.95 ± 0.15.

***GA cost function***. The root mean square error (*E*_*RMS*_) between τ^*p*^ - τ^*g*^ and τ^*m*^, has been selected as the cost function of the GA to be minimized:

(7) $$\begin{array}{l} CostFunction=\sqrt{\frac{\sum_{i=1}^{K}{\left(\tau_{i}^{m}-\left(\tau_{i}^{p}-\tau_{i}^{g}\right)\right)}^{2}}{K}} \end{array}$$

where *K* is the number of time samples composing the “*Training set*,” *i* is the index of the i-th time sample, τ^*p*^ is the total estimated torque by the NMS model, τ^*g*^ is the gravity torque estimated by the arm gravity model (see section 2.5.1) and τ^*m*^ is the interaction torque between the subject and the L-Exos computed using the tri-axial force sensor at the EE and the positional Jacobian (the superscript *m* stands for *measured*).

Table 2 reports the list of all the optimized muscle parameters with the corresponding bounds – the parameter with the apostrophe in the right column indicates the default value. Regarding the parameter *A* we adopted the boundaries reported in Buchanan et al. (2004), whereas, in order to let the NMS model to account for the limited number of muscles, we set the variation range of the maximum muscle force equal to $F_{O}^{m}$' × [0.7, 4]. The bounds of the other parameters were set as a percentage of variation of the default value. The default value of body mass parameters (*M*_*a*_, *M*_*fh*_) were set as percentages of body mass (Winter, 1990).

**Table 2:** Allowed variation range of each optimized NMS parameter.

**N^*o*^**	**Parameter**	**Bound**
1	*A*	[−3, 0[
2	$F_{O}^{m}$	$F_{O}^{m}$' × [0.7, 4]
3	*l*_*o*_	*l*_*o*_'±30%
4	σ_*A*_	σ_*A*_'±10%
5–8	$a_{i}^{l}$ of *l, i* = 1, …, 4	$a_{i}^{l}$'±30%
9–12	$a_{i}^{r}$ of *r, i* = 1, …, 4	$a_{i}^{r}$'±30%
13	*M*_*a*_	*M*_*a*_'±30%
14	*M*_*fh*_	*M*_*fh*_'±30%

*The parameter with the apostrophe indicates the default (or non-optimized) value*.

#### 2.5.3. Linear optimization procedure

The linear optimization method is based on the same set of equations used by the GA-based approach (Equations 2–6) but only considers the optimization of:

- the maximum muscle force $F_{O}^{m}$ to account for the limited number of modeled muscles;
- the mass of the modeled body segments due to its high variability.

This minimal set of optimized variables has been chosen for two main reasons: (1) to express the model in a form that is “linear in the parameters,” and (2) to adjust the two main macroscopic parameters that suffer from a high inter-subjects variability. The linear expression of the model can be easily obtained as follows.

Substituting the definition of *F*^*mt*^ (Equation 3) in the Equation 2, the total articulation torque τ^*p*^ can be expressed as follows:

(8) $$\begin{array}{l} \tau^{p}=\overset{N}{\underset{i=1}{\sum }}{F_{O}}_{i}^{m}A_{i},\;A_{i}=r_{i}\left(\theta \right)\cdot \left[{f_{l}}_{i}\left(\tilde{l_{i}}\right)\cdot a_{i}+{f_{P}}_{i}\left({\tilde{l}}_{i}\right)\right]\cos\phi_{i} \end{array}$$

In static conditions, the total torque exerted by a human articulation can be written as follow:

(9) $$\begin{array}{l} \tau^{h}=\tau^{g}+\tau^{e};\;\tau^{g}=\overset{J}{\underset{j=1}{\sum }}\tau^{g_{j}} \end{array}$$

where τ^*g*^ is the gravity torque applied by the considered articulation to sustain the distal part of the limb and τ^*e*^ is the torque applied to balance other external forces. The term $\tau^{g_{j}}$ represents the gravity contribution of the j-th limb link acting on the considered articulation. It is worth noting that in static conditions τ^*p*^ and τ^*m*^ are estimations of τ_*h*_ and τ_*e*_, respectively. Then it results:

(10) $$\begin{array}{l} \tau^{e}=\sum_{i=1}^{N}{F_{O}}_{i}^{m}A_{i}-\sum_{j=1}^{J}f_{j}^{sc}\tau \prime^{g_{j}} \end{array}$$

where τ′^*gj*^ is the non-optimized j-th gravity contribution and $f_{j}^{sc}$ is a scale factor that will be optimized in order to adjust the j-th gravity term (or, in other words, the mass of the j-th limb link). Since τ^*e*^ is expressed as a linear combination of parameters (Equation 10), it results that, if the terms *A*_*i*_ and τ′^*gj*^ are known, it is possible to optimize the coefficients ${F_{O}}_{i}^{m}$ and $f_{j}^{sc}$ using a linear optimization algorithm. The cost function to be minimized is equal to the cost function used in GA-based approach (see Equation 7).

### 2.6. Method comparison: optimization experiments

The performance of the two proposed optimization methods were evaluated using the datasets acquired as described in section 2.3. Firstly, the training and validation sets were created by randomly assigning each of the two isometric contractions (per direction and per point) to one set or to the other one. Each optimization method was then used to optimized both the shoulder and elbow articulations as two independent NMS models using the training dataset. The shoulder model includes the BB, TB, DA, and DP muscles, whereas the elbow model considers only the BB and TB muscles.

#### 2.6.1. GA-based optimization

The non-linear GA-based optimization employs *Genocop III* that is a genetic algorithm for constrained and unconstrained optimization (Michalewicz and Nazhiyath, 1995). The chromosome used for the shoulder's model optimization is composed by 50 genes (12 muscle parameters times 4 muscles plus 2 parameters of the gravity model-see Table 2), whereas the chromosome for the elbow's model optimization considers 25 genes (12 muscle parameters times 2 muscles plus 1 parameter of the gravity model-see Table 2). *Genocop III* was set with two different populations composed by 10 chromosomes each and with the following two stop criteria:

1. variation of the best fitness function value less than or equal to a certain threshold;
2. maximum number of generations reached.

#### 2.6.2. Linear optimization

Concerning the linear optimization of both shoulder and elbow models, we used the linear least squares algorithm. Focusing on the shoulder model, the equation 10 can be written as follow:

(11) $$\begin{array}{l} \tau_{sh}^{e}={F_{O}}_{-BB}^{m}A_{BB}+{F_{O}}_{-TB}^{m}A_{TB}+{F_{O}}_{-DA}^{m}A_{DA}+{F_{O}}_{-DP}^{m}A_{DP}\\ -f_{sh}^{sc}\tau_{g}^{sh}-f_{el}^{sc}\tau_{g}^{el} \end{array}$$

whereas the elbow model can be expressed with:

(12) $$\begin{array}{l} \tau_{el}^{e}={F_{O}}_{-BB}^{m}A_{BB}+{F_{O}}_{-TB}^{m}A_{TB}-f_{el}^{sc}\tau_{g}^{el}. \end{array}$$

### 2.7. Performance measures and statistical analysis

We compared the two proposed optimization methods evaluating the estimation performance on the validation dataset in terms of coefficient of determination (*R*^2^) and root mean square error (*E*_*RMS*_) between the measured and predicted torques. Differences in performance were assessed by a 3 × 2 repeated measures factorial design. The involved factors were the model optimization state (non-optimized, optimized with the GA-based method, optimized with the linear method) and the investigated articulation (elbow and shoulder). A 2-way ANOVA test was then conducted separately for each of the two performance metrics extracted (*E*_*RMS*_ and *R*^2^). The homogeneity of variance was assessed by means of the Mauchy test and, in case of significance, the Greenhouse-Geisser correction was adopted. Normality of the data was assessed by means of the Lilliefors test. Finally, we followed up the interaction in case of a significant interaction between the two factors.

## 3. Results

To address the question whether the GA-based method performs better than the linear one on a new dataset, i.e., validation dataset, the two methods were compared in terms of *R*^2^ and *E*_*RMS*_ between the moment predicted by the optimized model and the reference joint moments.

**Figure 2:**
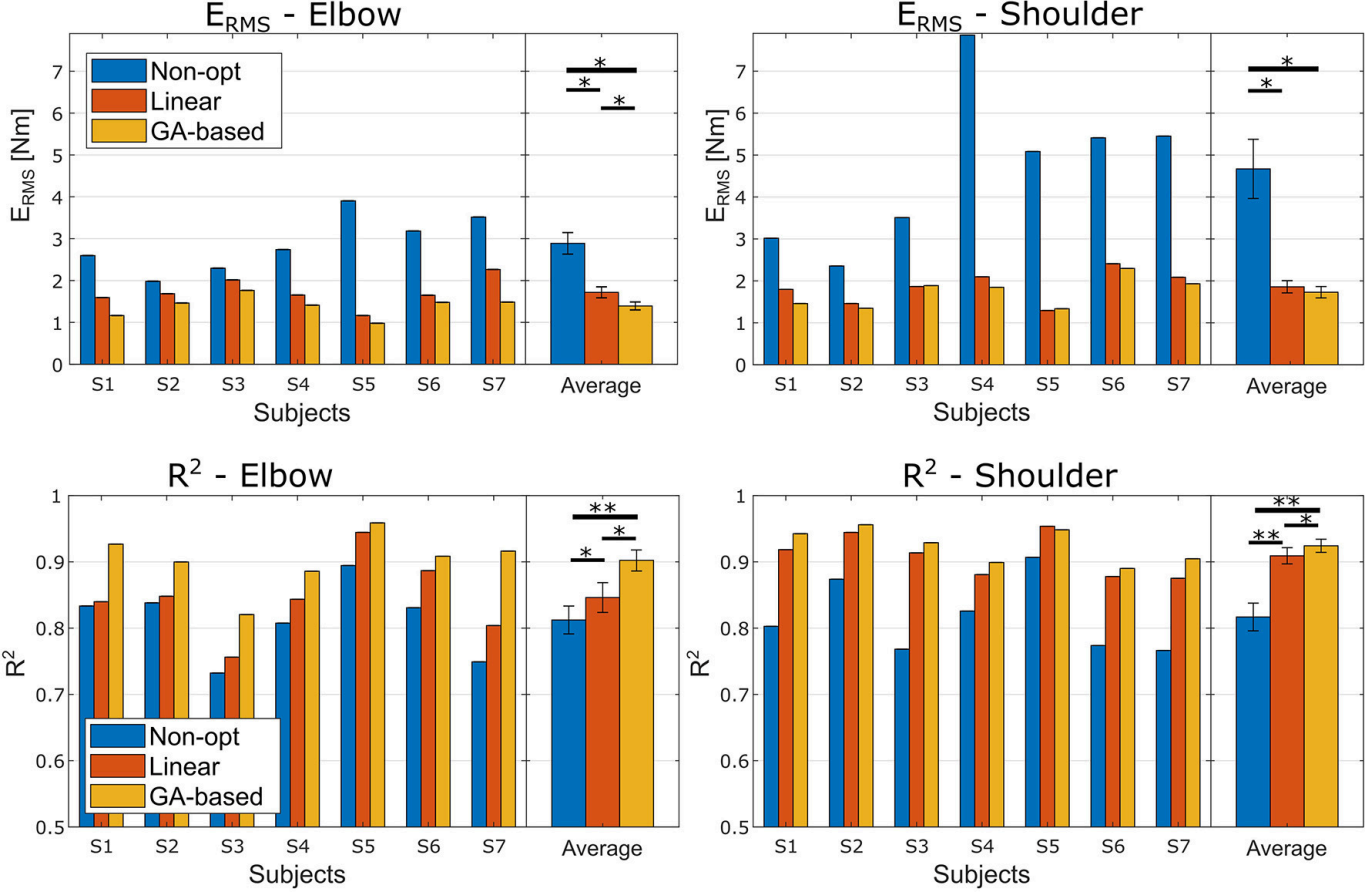
*E*_*RMS*_ and *R*^2^ performance obtained in the experiment. Left and right columns relate to the elbow and shoulder joints, respectively. The first row reports the *E*_*RMS*_ performance, and the second row the *R*^2^ performance. Each panel reports the performance for each subject and the averaged performance with the standard deviations. Asterisks mark the significance of the Bonferroni corrected *post-hoc* comparison tests (* < 0.05 and ** < 0.01).

Figure 2 reports the performance measures for each subject before the optimization and after the two different optimization procedures. The ANOVA test conducted using the *E*_*RMS*_ performance highlighted a high significant effect of the optimization method and a significant effect of the investigated articulation as well (Optimization method *F*_(2, 12)_ = 24.4, *p* < 0.001, $\eta_{p}^{2}=1.00$; Joint *F*_(1, 6)_ = 8.7, *p* = 0.026, $\eta_{p}^{2}=0.39$). There was also a significant ordinal interaction between the two factors, *F*_(2, 12)_ = 7.5, *p* =.008, $\eta_{p}^{2}=0.87$. Follow-up Bonferroni-adjusted pairwise comparison indicated that for both joints the two optimization methods lead to a significant better performance than the non-optimized models. Interestingly, as for the elbow joint we found that the error obtained with the Non-Linear optimization method was significantly lower than the one obtained with the Linear method (*p* = 0.034), the difference found for the shoulder joint was not significant (*p* = 0.140) even though the Non-Linear method lead to smaller *E*_*RMS*_.

Regarding the ANOVA test conducted on the *R*^2^ coefficients, we found a highly significant effect of the optimization method and, even though there was no effect of the Joint factor, a significant ordinal interaction effect was found (Optimization method *F*_(2, 12)_ = 50.8, *p* < 0.001, $\eta_{p}^{2}=1.00$; Joint *F*_(1, 6)_ = 3.6, *p* = 0.105, $\eta_{p}^{2}=0.36$; interaction *F*_(2, 12)_ = 9.2, *p* =.004, $\eta_{p}^{2}=0.93$). Follow-up Bonferroni corrected post-hoc tests, fixing the level of the Joint factor, revealed that, for both Joint factor's levels, the *R*^2^ obtained with the non-linear optimization method was significantly higher than the one obtained with the linear optimization method (*p* = 0.016 and *p* = 0.034 for elbow and shoulder, respectively).

Interestingly, fixing the level of the optimization state factor lead to different results regarding the two ANOVA tests conducted over the two performance metrics. In particular, the *E*_*RMS*_ on the shoulder joint was significantly higher than the elbow *E*_*RMS*_ for both the non-optimized model and the GA-based optimized model, whereas concerning the model optimized with the linear method there was no significant difference between the *E*_*RMS*_ of the two joints. The details about the *post-hoc* comparison are reported in Tables 3, 4 for the *R*^2^ and the *E*_*RMS*_, respectively.

**Table 3:** *R*^2^ performance *post-hoc* comparisons for each joint.

**Articulation**	**Opt. State**		**(I)**	**(J)**	***p*-value**
	**(I)**	**(J)**	**μ±σ**	**μ±σ**	
Elbow	No	GA	0.81 ± 0.06	0.90 ± 0.04	0.002
	No	Lin	0.81 ± 0.06	0.85 ± 0.06	0.015
	Lin	GA	0.85 ± 0.06	0.90 ± 0.04	0.016
Shoulder	No	GA	0.82 ± 0.06	0.92 ± 0.03	0.002
	No	Lin	0.82 ± 0.06	0.91 ± 0.03	0.001
	Lin	GA	0.91 ± 0.03	0.92 ± 0.03	0.034

**Table 4:** *E*_*RMS*_ performance Bonferroni corrected *post-hoc* comparisons for each joint.

**Articulation**	**Opt. State**		**(I)**	**(J)**	***p*-value**
	**(I)**	**(J)**	**μ±σ [Nm]**	**μ±σ [Nm]**	
Elbow	No	GA	2.89 ± 0.68	1.39 ± 0.25	0.010
	No	Lin	2.89 ± 0.68	1.72 ± 0.35	0.030
	Lin	GA	1.72 ± 0.35	1.39 ± 0.25	0.022
Shoulder	No	GA	4.67 ± 1.87	1.73 ± 0.36	0.012
	No	Lin	4.67 ± 1.87	1.86 ± 0.39	0.015
	Lin	GA	1.86 ± 0.39	1.73 ± 0.36	0.140

The durations of the linear and GA-based optimization procedures are 0.0010 ± 0.0005 s and 90 ± 30 s, respectively. All the tests have been run on a PC with Microsoft Windows7 64 bit, CPU quad core i7 1.73 GHz and 8 GB RAM.

## 4. Discussions

In this study we showed that the shoulder and elbow moment, generated along the sagittal plane in isometric conditions, could be predicted using a minimal upper limb NMS model optimized with a simple linear approach. Although the isometric condition is far from the natural functioning of the muscles in daily-life activities (Merletti and Farina, 2016), we considered and optimized the model in isometric conditions with the goal of reducing the model complexity, that is in line with the main rationale of the study. This choice has been done already in previous works (Manal et al., 2012; Buongiorno et al., 2016), in which a model optimized in isometric conditions was used in quasi-static conditions for the real-time estimation of joint torques. The linear optimization approach has been compared with a GA-based optimization one. The two optimization methods differed not only for the algorithm complexity, but also in terms of the number of optimized parameters.

As reported in Figure 2, both the proposed optimization methods were able to significantly improve the articulation moment estimation. As expected, these significant improvements clearly evidenced the need for an optimization procedure to adapt the model to the specific subject. In particular, considering both the proposed approaches together, the average *E*_*RMS*_ percentages of improvement due to the optimization methods were of 46.1 and 61.57% for the elbow and the shoulder, respectively, and the average *R*^2^ percentages of variation were of 7.6 and 12.23% for the elbow and the shoulder, respectively. Looking at Figure 2, it is interesting to note how both the optimization procedures lead to higher improvement for shoulder articulation than for the elbow articulation. This finding could be related to the fact that the proposed model for the shoulder is much more approximated than the adopted model for the elbow model. In fact, among the nine muscles crossing the elbow joint (nine muscles, i.e., Triceps Long, Triceps Lateral, Triceps Medial, Anconeus, Supinator, Biceps Long, Biceps Short, and Brachialis Brachioradialis) the biceps and the triceps could be easily considered the two main muscles. Moreover, the elbow joint could be considered an articulation with only one DoF. On the other hand, the shoulder can be considered as a 4 DoFs articulation (Seth et al., 2016) actuated by a multitude of muscles (18 muscles, i.e., Deltoid Anterior, Deltoid Middle, Deltoid Posterior, Supraspinatus, Infraspinatus, Subscapularis, Teres minor, Teres major, Pectoralis major Clavicular, Pectoralis major Sternal, Pectoralis major Ribs, Latissimus dorsi Thoracic, Latissimus dorsi Lumbar, Latissimus dorsi Iliac, Coracobrachialis, Biceps Long, Biceps Short, and Triceps Long) whereas the presented NMS shoulder model considered only four muscles. Hence, our model did not consider seven muscles of the elbow joint and 13 muscles of the shoulder joint (the Deltoid Middle should not give a contribution in flexion/extension in the sagittal plane) and it has to be noted that for each muscle there are a variety of parameters to be optimized.

Focusing on the comparison between the two optimization methods, an interesting aspect emerged following up the interaction of the ANOVA tests. Regarding the elbow joint, the GA-based method is significantly more effective in moment prediction than the linear method (*p* < 0.05 for both the *E*_*RMS*_ and *R*^2^ performance). This statement is not true for the shoulder model, where the difference between the two methods is not significant in term of *E*_*RMS*_ (*p* = 0.140). In fact, looking at the percentage of variation of *E*_*RMS*_ from the raw model, the GA-based approach reached an improvement of 51.78 and 61.57% for the elbow and the shoulder respectively, whereas the linear procedure obtained a percentage of variation of 40.51 and 60.19% for elbow and shoulder, respectively (see Figure 2). Thus, it can be noted that there is a difference between the two methods of 11.2 percentage points for the elbow vs. the 2.7 points for the shoulder. This is quite interesting since these findings could actually reveal that a deep optimization of the NMS model of a specific articulation is more effective if the modeled muscles are representative of the articulation. Hence, we could claim that, the use of the linear method to optimize a limited number of model parameters leads to similar performances achieved by optimizing a large set of parameters using a more complex and computationally expensive approach (GA-based). This result could also suggest a way for selecting the best approach between adjusting a large set or a small set of parameters based on the level of model simplification (in terms, for instance, of number of considered primary muscles).

The big computational time needed for calibrating a NMS model represents a well-known problem in literature (Sartori et al., 2012, 2009). In particular, a long optimization procedure limits the use of model-based approach in myo-control. As the optimization time using the linear method is smaller than the optimization time required by the GA-based method (milliseconds vs. few minutes), the similar results yielded by the two methods are particularly relevant for the possible final applications of the system, in which a short time spent for the training phase increases both the usefulness and the usability of the system. It is worth noting that an NMS model accounting for a high level of detail leads to long lasting optimization procedures with an average timing of 1 h (Pau et al., 2012).

A simple model could also potentially avoid the “over-fitting” problem. More in detail, it can be taken for granted that the more muscles are considered, the more parameters can be adjusted, and the more those parameters are allowed to change, the better the model fitting will be. However, as found by Zheng et al. (1998) and discussed by Buchanan et al. (2004), *“Too Many Parameters Is Not Good.”* In fact, if from one side having many parameters could potentially improve the fit between the estimated joint moment and the measured joint moment, from the other side models with many parameters generally have little predictive ability. However, as future works, it would be interesting to investigate how the torque estimation performance and the computational time of the optimization would change by incrementally increasing the model complexity in terms of both analyzed muscles and optimized parameters.

**Figure 3:**
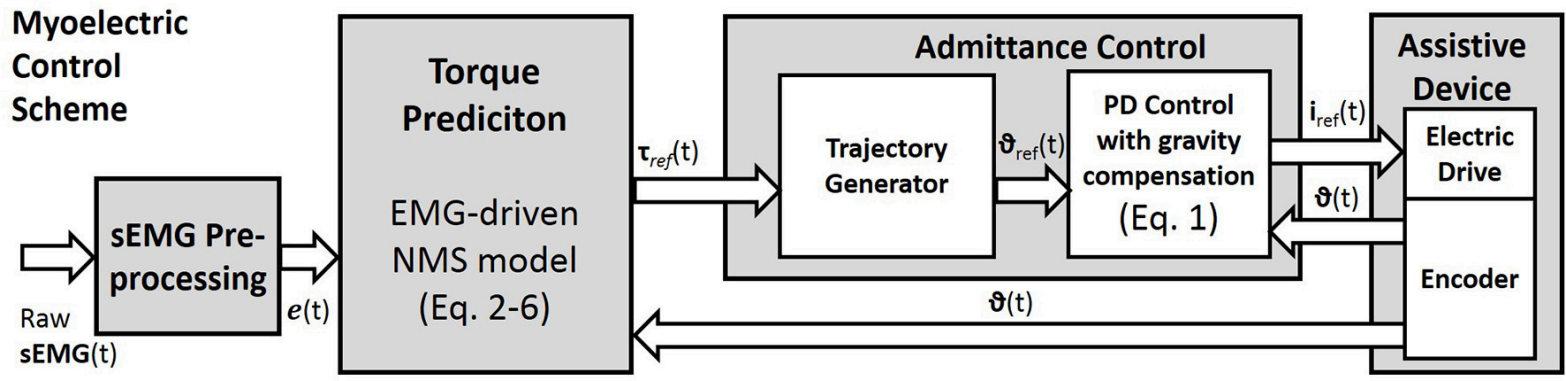
The myoelectric Control Scheme. Raw surface EMG signals are pre-processed to compute the muscle excitation e(t). The neuromusculoskeletal model is used to predict the reference torques (Equations 2–6). Through the admittance control the predicted torques are used to generate the reference joint angles of the assistive device. The local controller is based on a position PD controller with gravity compensation. The measured joint positions are fed back to the PD controller and to the torque predictor.

For future studies, the two proposed optimization techniques may be compared in dynamic conditions and tested within a myo-control scheme. However, we have previously tested both optimization methods in a myoelectric control scenario with promising results (Buongiorno et al., 2015, 2016). In particular, we proposed an admittance control able to generate the desired trajectory of an upper limb exoskeleton according to the EMG-based motion intention detection of the user (Figure 3). The described myoelectric control was assessed evaluating the measured interaction force between the subject and the exoskeleton while performing free movements at different speeds along the sagittal plane.

## 5. Conclusion

This paper has presented a linear optimization procedure to optimize an EMG-driven neuromusculoskeletal model of the shoulder and elbow that consider a reduced number of muscles. We compared the linear method with a complex approach that consider a genetic algorithm. The two methods differed both in the numbers of optimized parameters and in the optimization procedure. As expected, both the two methods substantially increased the estimation performance of the original model showing significant improvements in both the two explored arm joints. Thanks to the low computational cost and the short setup phase required for wearing and calibrating the system, obtained results are promising for being introduced in an industrial or rehabilitation scenario for intention recognition purposes. Future works will include the comparison of the two optimization approaches with other body articulations and in myo-control with admittance control of an upper-limb exoskeleton.

## Author contributions

DB, MB, VB, and AF conceived and designed the experiments, DB and FB performed the experiments, DB, FB and MB analyzed the data; DB, VB, and AF contributed to materials and analysis tools, DB and MB wrote the paper.

### Conflict of interest statement

The authors declare that the research was conducted in the absence of any commercial or financial relationships that could be construed as a potential conflict of interest. The reviewer FCl declared a shared affiliation, though no other collaboration, with several of the authors MB, FB, and AF to the handling Editor.
