# Supplementary material for: A Linear Approach to Optimize an EMG-Driven Neuromusculoskeletal Model for Movement Intention Detection in Myo-Control: A Case Study on Shoulder and Elbow Joints
Source: Front Neurorobot. 2018 Nov 13;12:74. doi: 10.3389/fnbot.2018.00074 (PMC6243090; doi:10.3389/fnbot.2018.00074)
Supplement: Supplementary file 1 [file Data_Sheet_1.PDF]

Supplementary Material: A linear approach to optimize an EMG-driven neuromusculoskeletal model for movement intention detection in myo-control: a case study on shoulder and elbow joints

1 EMG-DRIVEN NEUROMUSCULOSKELETAL MODEL

This section briefly describes the basic equations of an EMG-driven neuromusculoskeletal model (see (Buchanan et al., 2004) for more details).

1.1 The Hill-based muscle-tendon model

A muscle-tendon model is composed by a muscle fiber in series with a tendon represented by an elastic or viscous-elastic element. The muscle fibers are modeled as a Contractile Elements (CE) with a Passive Element (PE) in parallel. The CE generates the active muscle force (F_A^m) receiving the commands from the motor neurons. The PE, instead, is an elastic component that simulates the passive force (F_P^m) generated by the elasticity of the muscle tissue. In particular, the Hill-based muscle model (Hill, 1938) refers just to the contractile element. An EMG-driven Hill-based muscle-tendon model is able to estimate the force F^{mt} generated by a muscle-tendon unit using the simple equations that follow:

$$\begin{aligned} F^{mt} &= F^m \cos\phi \\ &= \left[F_A^m(\tilde{l}, v) + F_P^m(\tilde{l})\right] \cos\phi \\ &= F_O^m \left[f_l(\tilde{l}) \cdot f_v(v) \cdot a + f_P(\tilde{l})\right] \cos\phi \end{aligned} \tag{S1}$$

with,

$$F_A^m(\tilde{l}, v) = F_O^m \cdot f_l(\tilde{l}) \cdot f_v(v) \cdot a \tag{S2}$$

$$F_P^m(\tilde{l}) = F_O^m \cdot f_P(\tilde{l}) \tag{S3}$$

$$\phi = arcsin\frac{l_o \sin\phi_o}{l}, \qquad \tilde{l} = \frac{l}{l_o} \tag{S4}$$

where F^m is the force generated by the muscle fibers; $f_l(\tilde{l})$ is the normalized fiber length-force relationship; $f_p(\tilde{l})$ is the normalized fiber length-passive force relationship; $f_v(v)$ is the normalized fiber contraction velocity-force relationship; a is the muscle activation level; F_O^m is the maximum isometric muscle fiber force; l and v are the fiber length and fiber contraction velocity; $\tilde{l}$ is the normalized fiber length; l_o is the optimal fiber length; ϕ_o is the pennation angle at l_o. Regarding the physiological meaning of the variables, it is worth mentioning that: l_o is the muscle fiber length at which the muscle generates the maximum isometric force, F_O^m is the muscle force generated in maximum voluntary contraction condition and the muscle fiber length changes as the crossed articulation angle (θ) changes. In addition, in a detailed hill-based muscle model, the optimal fiber length l_o depends on the amount of muscle activation (Buchanan et al., 2004).

EMG-driven muscle activation extraction. The muscle activation is a signal that ranges from 0 to 1, where 0 refers to no activation and 1 indicates that the muscle is fully activated. The extraction of the EMG-driven muscle activation has been deeply studied in the literature (Buchanan et al., 2004) and considers two main muscle's properties: (1) the muscle activation dynamic and (2) the non-linear relation between EMG signals and muscle force. These two properties can be modeled with the following equations:

$$a(t) = \frac{e^{Au(t)} - 1}{e^{A} - 1} \tag{S5}$$

$$u(t) = \alpha e(t-d) - \beta_1 u(t-1) - \beta_2 u(t-2) \tag{S6}$$

with,

$$\beta_1 = \gamma_1 + \gamma_2, \qquad \beta_2 = \gamma_1 \times \gamma_2 \tag{S7}$$

$$|\gamma_1| < 1, \qquad |\gamma_2| < 1 \tag{S8}$$

$$\alpha - \beta_1 - \beta_2 = 1 \tag{S9}$$

where the parameter A identifies the non-linearity shape factor (A can range in $[-3, 0[$); d is the electro-mechanical delay (d can range from 10 ms to 150 ms Corcos et al. (1992)) and $e(t)$ is the pre-processed EMG signal. Such EMG signal processing usually accounts four consecutive steps:

1. high-pass filtering (20 Hz);
2. rectification;
3. normalization respect to the peak value of the rectified EMG obtained during the maximum voluntary contraction (MVC);
4. low-pass filtering (5-10 Hz).

The variables α, β_1 e β_2 are the coefficients that define the muscle activation dynamics. Thus, if γ_1 + γ_2 are known, β_1 and β_2 can be found from Equations reported at S7 and α can be found from Equation S9.

1.2 Musculoskeletal Model

The musculoskeletal model takes into account the position of both muscle's origin and muscle's insertion on the skeletal system. This geometry information is required to compute both the muscle fiber length with respect to the articulation angle and the muscle '*moment arm*' with respect to a specific articulation. The moment arm is needed to estimate the muscle's contribution to the total articulation's moment. In detail, the moment arm is the length of the segment that originates from the articulation center of rotation and ends perpendicularly on the tendon's line of action. The muscle moment arm r relative to one specific articulation can be described based on the displacements methods An et al. (1984), which is defined by:

$$r(\theta) = \frac{\partial l(\theta)}{\partial \theta} \tag{S10}$$

where θ is the articulation angle.

Given the values of force and moment arm of all muscles crossing a specific articulation, the total predicted moment applied at the articulation, i.e. (τ^p), is the sum of all single contributions as follows:

$$\tau^p = \sum_{i=1}^{N} \tau_i = \sum_{i=1}^{N} F_i^{mt} r_i(\theta) \tag{S11}$$

where N is the number of muscles acting on the modeled articulation.

REFERENCES

Buchanan TS, Lloyd DG, Manal K, Besier TF. Neuromusculoskeletal modeling: estimation of muscle forces and joint moments and movements from measurements of neural command. *Journal of applied biomechanics* **20** (2004) 367.

Hill A. The heat of shortening and the dynamic constants of muscle. *Proceedings of the Royal Society of London B: Biological Sciences* **126** (1938) 136–195.

Corcos DM, Gottlieb GL, Latash ML, Almeida GL, Agarwal GC. Electromechanical delay: an experimental artifact. *Journal of Electromyography and Kinesiology* **2** (1992) 59–68.

An K, Takahashi K, Harrigan T, Chao E. Determination of muscle orientations and moment arms. *Journal of biomechanical engineering* **106** (1984) 280–282.
